# The Genus *Pratylenchus* (Nematoda: Pratylenchidae) in Israel: From Taxonomy to Control Practices

**DOI:** 10.3390/plants9111475

**Published:** 2020-11-02

**Authors:** Patricia Bucki, Xue Qing, Pablo Castillo, Abraham Gamliel, Svetlana Dobrinin, Tamar Alon, Sigal Braun Miyara

**Affiliations:** 1 Volcani Center, Department of Entomology, Nematology and Chemistry Units, Agricultural Research Organization (ARO), Rishon Lezion 15159, Israel; pbucki@volcani.agri.gov.il (P.B.); xueqing4083@gmail.com (X.Q.); 2 Department of Plant Protection, Nanjing Agricultural University, Nanjing 210095, China; 3 Institute for Sustainable Agriculture, Spanish National Research Council, 14004 Cordoba, Spain; p.castillo@csic.es; 4 Volcani Center, Laboratory for Pest Management Research, Institute of Agricultural Engineering, ARO, Rishon Lezion 15159, Israel; agamliel@volcani.agri.gov.il; 5 Extension Service (Shaham), Israel Ministry of Agriculture and Rural Development, Rishon Lezion 15159, Israel; svetyd@gmail.com (S.D.); tamalon6@gmail.com (T.A.)

**Keywords:** *Pratylenchus*, root lesion nematode, pathogenicity, distribution, molecular phylogeny, taxonomy, control management practices

## Abstract

Due to Israel’s successful agricultural production and diverse climatic conditions, plant-parasitic nematodes are flourishing. The occurrence of new, previously unidentified species in Israel or of suggested new species worldwide is a consequence of the continuous withdrawal of efficient nematicides. Among plant-parasitic nematodes, migratory endoparasitic species of the genus *Pratylenchus* are widely distributed in vegetable and crop fields in Israel and are associated with major reductions in quality and yield. This review focuses on the occurrence, distribution, diagnosis, pathogenicity, and phylogeny of all *Pratylenchus* species recorded over the last few decades on different crops grown throughout Israel—covering early information from nematologists to recent reports involving the use of molecular phylogenetic methodologies. We explore the accepted distinction between *Pratylenchus thornei* and *Pratylenchus mediterraneus* isolated from Israel’s northern Negev region, and address the confusion concerning the findings related to these *Pratylenchus* species. Our recent sampling from the northern Negev revealed the occurrence of both *P. thornei* and *P. mediterraneus* on the basis of molecular identification, indicating *P. mediterraneus* as a sister species of *P. thornei* and their potential occurrence in a mixed infection. Finally, the efficiencies of common control measures taken to reduce *Pratylenchus*’ devastating damage in protected crops and field crops is discussed.

## 1. Introduction

Root-lesion nematodes of the genus *Pratylenchus* are migratory endoparasites belonging to the family Pratylenchidae (Nematoda, Tylenchina), with around 100 species recognized today [1,2,3]. *Pratylenchus* species can cause yield losses of up to 85% of expected production [4], and even higher losses when nematodes interact synergistically with certain soilborne plant pathogens [5]. Hence, *Pratylenchus* species are highly relevant to agriculture.

Israel is a small semiarid country located in western Asia, only 22,000 km^2^ in size. Despite the fact that the geography of the country is not naturally conducive to agriculture, advanced irrigation, cultivation mastery, use of elite varieties, and the introduction of state-of-the-art agricultural technologies contribute, in practice, to intensive and efficient farming. On the other hand, this success in agricultural productivity along with a diversity of climatic conditions have led to the proliferation of devastating plant-parasitic nematodes. Among them, *Pratylenchus* species are widely distributed in vegetable and crop fields in Israel and are associated with a major reduction in quality and yield. The genus *Pratylenchus* was first reported from Israel in 1957 [6]. Since then, several studies related to this nematode have been published [7,8,9,10,11,12,13,14]. However, these studies are largely scattered. Some of them are published in less accessible local journals, such as master’s or PhD theses, or in scientific reports written in Hebrew. In this review, we collected all available information on *Pratylenchus* in Israel, spanning the last few decades, from local Hebrew journals to international peer-reviewed ones, revealing that *Pratylenchus* species are major pests in many crops throughout the country. We provide a comprehensive summary of the occurrence, taxonomy, distribution, diagnosis, and pathogenicity of *Pratylenchus* in Israel, along with an overview of the status and perspectives for *Pratylenchus* research in this country.

## 2. Overview of Israeli Agriculture

Agriculture is an important sector for the Israeli economy, representing around 2.5% of Israel’s GDP and about 3.5% of its exports. Agricultural production is especially significant in certain areas, such as the Arava, Jordan Rift Valley, and northern Negev, where it provides almost the sole means of livelihood for the population. Although some of these regions are characterized by semiarid land with varied climatic [15,16], topographical, and soil conditions, determination and farming ingenuity have produced maximum yields and crop quality [17]. Among the most common agricultural sectors, vegetable growing has become a specialized skill of Israeli farmers, on the basis of selecting suitable hybrid varieties, fertilizers, irrigation methods, greenhouse covers designed for specific crops, innovative growing tools, and plant protection management. Moreover, vegetable growing exploits the sunshine and high temperatures, providing high-quality vegetables during the competitors’ off seasons in other countries. As a result, vegetables account for about 17% of Israel’s total crop output value. About two-thirds of Israel’s field crops are grown on non-irrigated land. These rain-fed crops include wheat for grains, silage and hay, legumes for seeds, and sunflower for oil. The remaining field crops are summer crops, including cotton, chickpeas, green peas, beans, corn, groundnuts, and watermelon for seeds, most of which are irrigated. Fruit trees mainly include deciduous fruit orchards that are among the main crops in northern Israel, including grapevine, fig, almond, apple, pear, stone fruit, pomegranate, and persimmon, as well as subtropical varieties (citrus, avocado, mango, olive, litchi) and small fruit (various berries).

## 3. Occurrence of *Pratylenchus* Species in Israel

The most comprehensive survey of Israel’s soil nematodes was performed by Cohn et al. [7], wherein 320 soil samples were taken from natural agro-ecosystems, providing a backbone for soil nematode diversity and distribution in Israel (Table 1, Cohn et al. [7]). This survey suggested that in cultivated crops grown throughout Israel, *Pratylenchus* species were among the three most prevalent plant-parasitic nematodes infecting vegetables (49% of the samples, see below), cereal and pasture grasses (68%), pasture legumes (48%), and deciduous fruit trees (47%). Less commonly, *Pratylenchus* species were found in natural vegetation fruit trees (35%) and forest trees (30%), and in cultivated crops of subtropical and tropical fruit trees (20%), grapevines (29%) and lawns (27%) (Table 1). Geographically, *Pratylenchus* was most prevalent in the Negev, located in southern region of the country (54%), while its abundance in the rest of the geographical locations ranged between 33 and 49% [7].

## 4. Taxonomy and Diversity of *Pratylenchus* Species in Israel

The first species of *Pratylenchus* were identified by Minz in 1957 [6]: *Pratylenchus brachyurus* (Godfrey, 1929), *Pratylenchus neglectus* (=*P. minyus*) (Rensch, 1924), *Pratylenchus penetrans* (Cobb, 1917), and *Pratylenchus scribneri* (Steiner in Sherbakoff & Stanley, 1943). Later, Cohn et al. [7] added three more species: *Pratylenchus pratensis* (de Man, 1880), *Pratylenchus thornei* (Sher and Allen, 1953), and *Pratylenchus vulnus* (Allen & Jensen, 1951), and Corbett (1983) described a new species, *Pratylenchus mediterraneus*. Most recently, Qing et al. [18] added another new species, *Pratylenchus capsici*.

**Figure 1:**
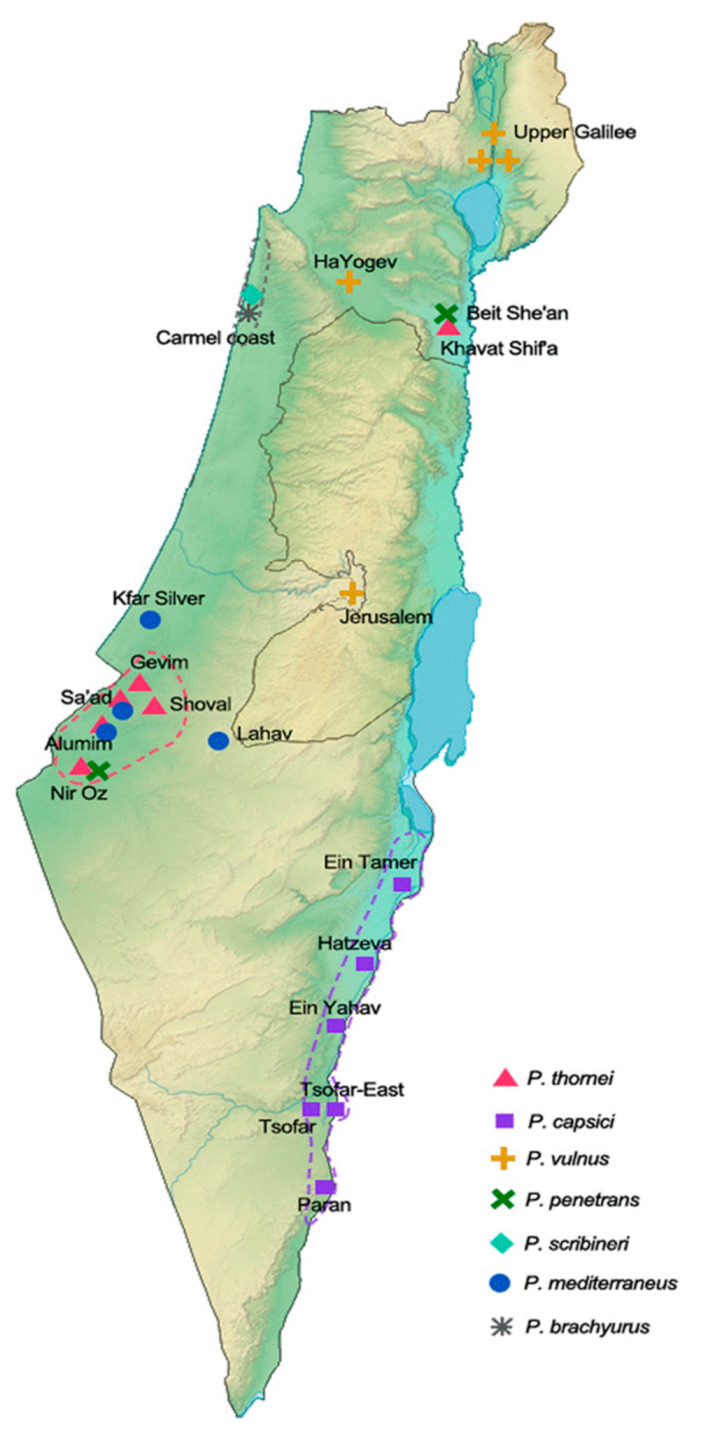
Map of the known distribution of *Pratylenchus* species recorded in Israel’s farming regions. Only recorded infested regions are indicated for each *Pratylenchus* species.

To date, nine species of *Pratylenchus* have been reported throughout the country (Figure 1). Most of these species have only been identified by morphological characteristics, but three of them have been recently confirmed by molecular data. Their distribution and associated plant hosts are detailed below.

### 4.1. Pratylenchus mediterraneus Corbett, 1983

Orion et al. [19] and Krikun and Orion [9] observed an unusual population of *P. thornei* parasitizing potatoes in the northern Negev. After a detailed morphological and morphometric study, Corbett and Clark [20] designated this population as a new species, *P. mediterraneus*. Although the validity of *P. mediterraneus* designation was questioned [21], it is generally accepted as a valid species [1,2,22,23], being further supported by a variety of molecular evidence, such as restriction fragment length polymorphism (RFLP) analysis of ribosomal (r)DNA fragments [24,25,26], sequences of rDNA D3 expansions [27], and sequences of 18S and 28S rDNA [18]. Morphologically, *P. mediterraneus* is closely related to *P. thornei* in labial region shape en face pattern, and only differs in having a shorter stylet, sexual reproduction, and males being common [28]. Therefore, the identities of several *P. thornei* populations reported from various Middle Eastern countries [29,30] are suspected to be *P. mediterraneus*. The matrix code for *P. mediterraneus* is A2, B2, C2, D2, E2, F3, G2, H1, I3, J1, K1 ([23]; Supplementary Material Table S1; Supplementary Material File S2).

*Pratylenchus mediterraneus* was originally found in the northern Negev region of Israel [14,19]. Later, this species was recorded on chickpea in Turkey [31,32]; chickpea and lentil in Syria [33,34]; legumes in Algeria, Tunisia, and Morocco [33,35]; and chrysanthemum in Korea [36]. In Israel, *P. mediterraneus* primarily parasitizes legumes and cereals, which are the prevalent crops in the northern Negev, but carrot and potato can also be hosts [37]. Hosts reported by the Plant Protection and Inspection Services (PPIS) of the Israeli Ministry of Agriculture and Rural Development currently include alfalfa, barley, beans, broad beans, cabbage, carrot, chickpea, clover, coriander, lovage, sweet potato, vetch, and wheat [38].

### 4.2. Pratylenchus thornei Sher and Allen, 1953

In Israel, *P. thornei* has been reported on potato [13], cereals such as wheat and barley [8,10,19], carrots [37], legumes such as *Vicia sativa*, alfalfa and trifolium [39], watermelon [19], and cabbage [38], all in the northern Negev. However, most of these are likely to be *P. mediterraneus* [13,14,37]. Given the similarity between these two species, and the fact that *P. thornei* can occur in a wide range of soil types and is commonly found in mixed populations [39,40], the existence of *P. thornei* as part of a mixed population alongside *P. mediterraneus* in these studies is suspected. Notably, 28S rDNA-based molecular identification in recent samplings (2018 and 2019) has suggested the wide distribution of *P. thornei* in barley fields in Gevim, Alumim, and Nir-Oz located in the northern Negev (Table 2, Figure 1), and wheat fields in the Khavat Shif’a region and Avuka (Bet Shean Valley) located in the north of Israel [41].

This confirms the presence of *P. thornei* but fails to support the co-existence of *P. mediterraneus* and *P. thornei* within the same field populations. Diagnostic parameters described a labial region with three annuli, not offset from the body, an outer margin of sclerotized labial framework extending conspicuously around two annuli into the body, and one annulus into the labial region; lateral fields with four lines—the outer ones straight or weakly crenate; medium-length stylet (17–19 µm), a spermatheca that is difficult to see and does not contain spermatozoa; and males being very rare. The matrix code for *P. thornei* is A2, B2, C3, D1, E2, F2, G3, H1, I3, J1, K1. According to Castillo and Vovlas [23], it can be distinguished from the closely related species *P. penetrans* and *P. mediterraneus* by labial region shape, stylet length, the low proportion of males, and spermatheca and tail shapes.

### 4.3. Pratylenchus neglectus (Rensch, 1924) Filipjev and S. Stekhoven, 1941

In Israel, *P. neglectus* was first recorded by Minz [6] in association with fig tree roots. It is also known as the California meadow nematode, and has been reported by the Israeli Society of Plant Pathology (ISPP) on cotton crops and fig trees [38]. *Pratylenchus neglectus* is characterized by a labial region with two annuli, the second annulus wider than the first, anteriorly indented stylet knobs, a post-vulval uterine sac that is less than or equal to the body diameter, a variably shaped tail that is usually conoid with a little curvature of the ventral surface, and a tail terminus without annulation that is usually rounded but may be obliquely truncate or slightly digitate [23]. The matrix code for *P. neglectus* is A1, B2, C3, D1, E2, F1, G3, H1, I1, J1, K1.

### 4.4. Pratylenchus vulnus Allen and Jensen, 1951

*Pratylenchus vulnus* was first recorded by Cohn et al. [7] It is reported to be the most frequently encountered nematode associated with several pome and stone fruit trees, e.g., cherry, pear, plum, olive, apricot, nectarine, mango, persimmon, almond, citrus, fig, peach, and avocado, as well as some ornamentals including roses [38]. It is frequently found in rose nurseries, as well as in loquat, stone fruit, and apple trees in the north of Israel, very often in dense populations [42].

*Pratylenchus vulnus* is characterized by a labial region that is almost continuous with the body contour, with three or four annuli, a pharynx overlapping the intestine ventrally in a long lobe, an oblong spermatheca, a post-vulval uterine sac that is around two vulval body diameters long with a rudimentary ovary, and a tapering tail with a narrowly rounded subacute smooth tip; males are common. The matrix code for *P. vulnus* is A2, B2, C2, D3, E2, F6, G3, H3, I2, J1, K1.

### 4.5. Pratylenchus pratensis (de Man, 1880) Filipjev, 1936

*Pratylenchus pratensis* was first recorded by Cohn et al. [7], being described as *Anguillulina pratensis*. *Pratylenchus pratensis* has been found on Chinese cabbage, turnip, cauliflower, kohlrabi, white cabbage, radish, and cabbage by the ISPP [38]. This nematode species is characterized by a finely annulated cuticle, a labial region with three annuli, an oval to rectangular spermatheca, a post-vulval uterine sac length similar to the body diameter, and a tail with 20–28 annuli that are annulated to the terminus [23]. The matrix code for *P. pratensis* is A2, B2, C2, D4, E2, F3, G3, H2, I1, J1, K1. This species can be differentiated from closely related species by stylet length, the position of the vulva, shape of the spermatheca, shape of the tail, tail annuli, tail tip, and the presence of males.

### 4.6. Pratylenchus capsici Qing, Bert, Gamliel, Bucki, Duvrinin, Alon, Braun-Miyara, 2019

*Pratylenchus capsici* is an endemic Israeli species that has been recently identified from the roots of pepper [18], currently its only known host, with substantial damage observed. With the type population recovered from Tsofar farm, this species is widely spread across the pepper-growing region in the Arava Rift Valley of Israel. *Pratylenchus capsici* has been shown to be a cryptic species of *Pratylenchus oleae*, as they are almost indistinguishable morphologically. In fact, in the tabular key for *Pratylenchus* species identification proposed by Castillo and Vovlas [23], 10 out of 11 traits were identical for the two species. However, *P. capsici* differs from *P. oleae* in several molecular markers, as well as by several minor morphological differences, including the presence of males in the former, a functional spermatheca (vs. nonfunctional and empty in the latter), a larger body (559–642 for *P. capsici* vs. 412–511 μm for *P. oleae*), and a shorter stylet (14–15 vs. 15–17 μm, respectively) [18]. The matrix code for *P. capsici* is A2, B2, C2, D2, E1–3, F4–5, G2–3, H2, I1–2, J1, K2.

### 4.7. Pratylenchus penetrans (Cobb, 1917) Filipjev and Stekhoven, 1941

*Pratylenchus penetrans* was first recorded by Minz [6] in soil from a banana plantation. The ISPP has reported this species on lily, olive, nectarine, buttercup, apple, ruscus, strawberry, and peach [38]. It is also associated with grasses, cereals, and potatoes [42]. It was associated with the decline in pepper plants in the last decade in the Arava in a study carried out from 2004–2007, aimed at elucidating the causal agent of pepper collapse in that region [43]. Later on, *P. penetrans* continued to be identified in other studies as well [44]. Notably, during our intensive sampling of the Arava Rift Valley, *P. capsici* was the only root-lesion nematode associated with pepper. Given that *P. capsici* is morphologically similar to *P. penetrans* and species identification in these studies relied solely on morphology, here we consider that *P. penetrans* reported from the Arava might be *P. capsici*. Further morphology and molecular analyses are needed to confirm the distribution and host range of the former species. *Pratylenchus penetrans* is characterized by a labial region that is slightly offset, low, and flat in front with rounded outer margins, with three annuli; a pharynx overlapping the intestine ventrally; a lobe of around 1.5 body diameters in length; a short, undifferentiated post-vulval uterine sac, and a tail that is generally rounded with a smooth tip. The matrix code for *P. penetrans* is A2, B2, C3, D2, E3, F4, G2, H1, I3, J1, K1. It can be distinguished from closely related species by body and stylet length, number of lip annuli, labial framework, position of the vulva, and shape of the spermatheca and tail terminus [23].

### 4.8. Pratylenchus scribneri Steiner in Sherbakoff and Stanley, 1943

*Pratylenchus scribneri* was first recorded by Minz [6] in soils of banana, fig, plum, and quince trees. It has also been found on strawberry by the ISPP [38]. According to ISPP nematologists, *Pratylenchus* occurrence in banana plantations throughout Israel is very sparse [42].

This species is characterized by a labial region with two annuli that is slightly offset from the body, a stout stylet with rounded knobs, a pharyngeal overlap of medium length, an oblong spermatheca, and a slightly tapering tail with a smooth terminus. The matrix code for *P. scribneri* is A1, B2, C2, D3, E2, F4, G3, H1, I2, J1–3, K1.

### 4.9. Pratylenchus brachyurus (Godfrey, 1929) Filipjev and Stekhoven, 1941

*Pratylenchus brachyurus* was first recorded by Minz [6] and was found associated with other nematodes in soil from Cavendish banana. This species has also been reported on citrus [38].

This species is characterized by a labial region with two annuli, the anterior one showing an angular contour; a stylet with stout, rounded basal knobs; a vulva that is 82–89% of the body length; a post-vulval uterine sac that is less than one body diameter long; an inconspicuous nonfunctional spermatheca; and a tail that is broadly conoid, smooth, and broadly rounded, and truncate or spatulate at the tip. Males are rare. The matrix code for *P. brachyurus* is A1, B2, C4, D1, E4, F3, G3, H1, I4, J2–3, K1.

## 5. Biology and Pathogenicity of *Pratylenchus* Species

*Pratylenchus* species are polyphagous, migratory root endoparasites, developing and reproducing in the soil or roots. Their life cycle is simple and direct. The female lays its eggs singly or in small groups in the host root or in the soil near the root surface. Although little information is available about the true length of the *Pratylenchus* life cycle, on the basis of laboratory observations, research has estimated it to last from 45 to 65 days [45]. Symptoms caused by *Pratylenchus* are variable and depend on the host; they can include stunted and inefficient plant growth with reduced numbers of tillers and yellowed leaves.

Pathogenicity studies indicate that *Pratylenchus* species are very well adapted to parasitism, as extremely high populations in the soil do not kill their host plants. Nevertheless, damage thresholds range from 0.05 to 30 nematodes/cm^3^ of soil. Apart from direct damage to the roots, *Pratylenchus* species may also predispose plants to other pathogens (e.g., *Verticillium* and *Fusarium*), thereby increasing the damage extent [46,47]. Consequently, elimination of the nematode or reduction of its population causes a marked reduction in the incidence of fungi and an increase in crop yield. In Israel, the synergistic relationship between *P. thornei* and the fungus *Verticillium dahliae* caused a significant increase in the populations of both pathogens and in their damage to potato crops in the northern Negev [48].

Among the nine species recorded in Israel, *P. mediterraneus*, *P. thornei*, and *P. capsici* have been relatively more studied, and their biology and pathogenicity are discussed below.

*Pratylenchus mediterraneus* parasitism occurs mainly in the winter, but the nematode can survive for 7–8 months in a state of anhydrobiosis during the hot and dry season [8,49]. It is reactivated by the subsequent winter rains. In a field observation conducted by Orion et al. [10] from 1974 to 1983, the highest population of *P. mediterraneus* (as *P. thornei* in their paper) was recorded in the drought of 1978 and partial drought of 1982, and the lowest population in the unusually wet years of 1980 and 1983. Moreover, nematode populations with auxiliary irrigation treatments were extremely low. These data suggest that low moisture level—the natural condition in the northern Negev region—is a major ecological factor required for *P. mediterraneus* to build up its population, supporting the notion that *P. mediterraneus* is native to the semiarid zones of the Middle East [8,19] or, more specifically, the eastern Mediterranean region [50]. During the long hot season (April–November), the nematode population level remains stable due to anhydrobiosis [8]. In this state, the nematode can withstand conditions of 0% relative humidity, and desiccated nematodes can withstand temperatures of up to 40 °C. This characteristic enables their survival and facilitates their field or regional transmission in the northern Negev, where soil temperatures typically reach 40 °C in the hot season. This species is also likely to require the higher temperatures found in the Mediterranean region for its development, but this needs to be further studied.

In contrast to *P. mediterraneus*, the optimal temperature for *P. thornei* reproduction seems to be lower, ranging between 20 and 25° C [51,52], suggesting that the northern Negev may not be a suitable area for its survival. However, our molecular- and morphological-based analyses suggested that *P. thornei* is present not only in the mild northern Israel (Mesilot, Avuka, Shif’a), but also in the hot and dry region of the northern Negev [41]. In comparison, *P. mediterraneus* was only recovered from the northern Negev, suggesting that *P. thornei* may be able to adapt to a wider range of environmental conditions than *P. mediterraneus,* with the latter being more specialized for the hot and dry northern Negev.

The pathogenic effect of *P. mediterraneus* is limited to the early plant stages, resulting in reduced foliage and root growth of cereals and legumes, and thus influencing final plant density at harvest [12,14]. *Pratylenchus mediterraneus* was shown to be most concentrated in the root-tip region of hosts *Vicia sativa* and *Trifolium alexandrinum*. A histopathological study using scanning electron microscopy (SEM) showed nematodes penetrating the root epidermis and the cortical parenchyma through a clean-cut hole, probably a result of enzymatic activity and mechanical force [53]. When passing through parenchyma cells, *P. mediterraneus* can consume the cell contents, and these cells are thus void of cytoplasmic structures compared to the prominent nuclei and cytoplasmic structures in adjacent intact cells [12]. Typical symptoms caused by *P. mediterraneus* on common vetch were lesions produced along roots. These lesions lacked root hairs, with necrotic epidermal cells consisting of many holes, leading to severely deformed roots. Similar to *P. penetrans* [54], Orion and co-workers [12,37] speculated that *P. mediterraneus* can infect root tips as ectoparasites as well. Further SEM analysis showed the collapse of the parenchyma cells in the root lesion as the result of nematode feeding activity. The observed destruction was limited to the root cortex with an intact central cylinder, while nematode egg deposition was observed in cavities of the root cortex. These findings were similar to observations of *P. vulnus* in sour orange [55], *P. penetrans* in broad beans [56], and *Pratylenchus zeae* in maize [57].

*Pratylenchus penetrans* and *P. crenatus* Loof, 1960 have been reported worldwide as the major causal agent of carrots and Kuroda-type carrots [58,59,60,61]. In an investigation of carrot nematodes in Shoval, located in the northern Negev, we failed to detect these species. Instead, the field was infested with *P. thornei*, resulting in significant quality loss due to forking of carrot taproots [41]. However, whether *P. thornei* is the causal agent of these symptoms still needs to be confirmed, as continuous sampling from carrots demonstrated that the forking symptoms were not necessarily related to nematode occurrence [41].

**Figure 2:**
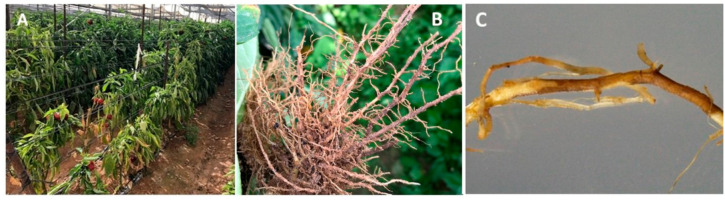
Symptoms caused by *Pratylenchus capsici*. (**A**) Pepper plant decline in the Arava Rift Valley characterized by stunted growth and wilting. (**B**) Heavily infected roots, with pronounced lesions along primary and secondary roots. (**C**) Photograph of developed root lesion taken under a dissecting microscope.

*Pratylenchus capsici* is an endemic Israeli species that is widely distributed in the Arava Rift Valley, causing significant yield reduction of pepper (Figure 2).

**Figure 3:**
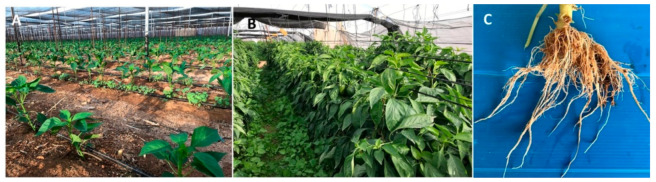
Weed distribution and function as a reservoir for *Pratylenchus capsici* during and in between growing seasons. (**A**) Weeds emerging early after pepper seedling planting, and (**B**) throughout the pepper-growing season. (**C**) Lesions caused by *P. capsici* on *Chenopodium album* growing alongside the pepper plants.

The emergence of this species was surprising, as this remote region is isolated from the country’s other agricultural areas. Moreover, until 1995, the entire region was free of reported nematodes, mainly due to intensive soil fumigation with methyl bromide [62]. Since the phase-out of this fumigant, certain species of *Meloidogyne* and *Pratylenchus* have become established in the soil, causing substantial damage to vegetable crops. Further biogeographical analysis suggested that a *P. capsici* population in weeds (*Chenopodium album* and *Sonchus oleraceus*) was an important source for *P. capsici* dispersal, either as the original nematode source or in maintaining the population between growing seasons (Figure 3).

Similar findings were observed for *P. penetrans* [63], *P. brachyurus* [64,65], *Pratylenchus coffeae* [66], *P. zeae* [67], *P. scribneri* and *P. vulnus* [68], and *P. thornei* and *P. neglectus* [69].

*Pratylenchus capsici* has been shown to survive through the seasons with no host from April to July. During this period, nematode activation is prevented by the high temperature and low moisture in the soil. Extensive nematode extraction from roots and soils yielded a high number of nematodes in the former and low numbers in the latter, supporting its exclusive endoparasitic life strategy. Therefore, these observations raise the question of whether *P. capsici* is ever anhydrobiotic, and if so, whether it goes through anhydrobiosis in the roots or in the soil. Similarly, *P. capsici*’s capacity to migrate to lower soil levels during the off seasons is not known. Further study is needed to clarify this question.

## 6. Phylogeny and Evolution of *Pratylenchus* Species Occurring in Israel

**Figure 4:**
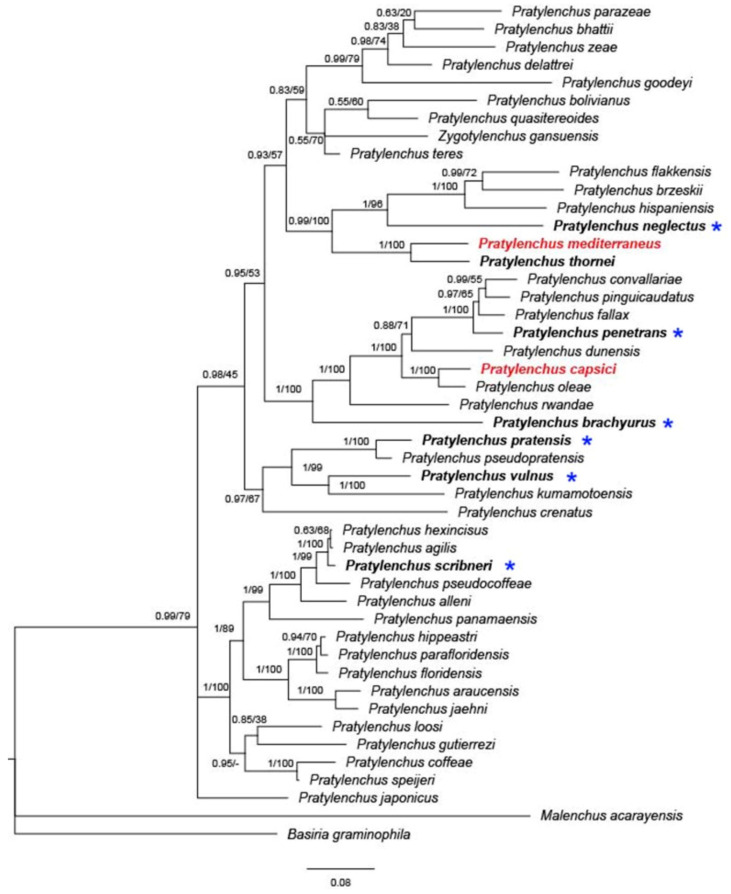
Bayesian 50% majority rule consensus tree inferred on concatenated sequences of 28S; asterisks indicate species that were only identified by morphology. The dataset was aligned by MAFFT v. 7.205 [70] using the G-INS-i algorithm. The phylogeny was reconstructed by maximum likelihood (ML) and Bayesian inference (BI) using RAxML v.8.1.11 [71] and MrBayes 3.2.3. [72]. Branch support is indicated in the following order: posterior probability (PP) value from BI analysis/bootstrap (BS) value from ML analysis. Red marked species indicate local Israeli isolates.

To date, nine species of *Pratylenchus* have been reported from Israel, with molecular data available for only three of them (*P. thornei*, *P. mediterraneus*, and *P. capsici*) (Figure 4). The concatenated phylogeny based on 18S and 28S rDNA and internal transcribed spacer (ITS) suggests that *P. thornei* and *P. mediterraneus* form a well-supported (posterior probability (PP) = 1, bootstrap (BS) = 100) monophyletic group, concurring with previous studies [18,24].

Orion [50] suspected that *P. mediterraneus* is a native or at least old inhabitant of the semiarid region of the Eastern Mediterranean. Given the similarities in morphology and morphometric features, the overlapping geographical area (Mediterranean region), the same hosts (mostly cereal and legumes), and the anhydrobiotic survival properties, *P. thornei* and *P. mediterraneus* could be derived from recent speciation events, with insufficient time to attain complete morphological differentiation.

Similarly, *P. capsici* is sister to *P. oleae* in concatenated phylogeny (Figure 4, PP = 1, BS = 100), as well as in a previous study [18]. *Pratylenchus oleae* was found in the Mediterranean region, parasitizing both wild and cultivated olive trees in Spain and Tunisia, with the presence of the nematode in wild olive not showing any clear symptoms in the aboveground plant or roots [3]. Interestingly, *P. capsici* was found in both pepper and weeds, markedly damaging the pepper but causing very mild symptoms on the weeds. Later, population genetic analysis revealed that *P. capsici* is likely to have been native to wild grass and transmitted to pepper by a recent expansion [18]. The adjacent distribution, similar morphology and presumably similar transmission background give rise to the idea that the two closely related species, *P. capsici* and *P. oleae*, may be native to the Mediterranean region.

## 7. Control and Management Practices

Plant growth and yield losses in any nematode–plant interactions depend primarily on soil nematode densities at planting. In the last few decades, intensive studies in Israel have been dedicated to the development of systems-based approaches to reducing soilborne pathogen densities at planting in different climatic regions [73,74,75,76,77]. These studies have shown that soil fumigants with nematicidal properties can reduce nematode infestation level but fail to eradicate the soil nematode, whereas a combination of fumigants with solarization can enhance the killing of soilborne pathogens [73,78,79], emphasizing the importance of using an appropriate combined application of pesticides and solarization.

### 7.1. The Use of Soil Fumigants

Three commercial soil fumigants are registered and commonly used in Israel: (i) 1,3-dichloropropene (1,3-D), a liquid fumigant (boiling point 104–112 °C) that is considered to be highly effective against nematodes and has been adopted as an alternative to methyl bromide [80]; 1,3,-D is registered for use in the control of all plant-parasitic nematodes and bacterial plant diseases, insects, and weeds. In practice, nematodes are the main target of 1,3-D use on most crops; 1,3,-D is labeled as a pre-planting soil treatment, and its effectiveness is dependent on environmental factors such as length of the growing season, moisture, temperature and soil type. (ii) Metam sodium (sodium N-methyldithiocarbamate, metam-Na) is widely used to control soilborne plant pathogens, mainly fungi and weeds, while its efficacy in the control of plant-parasitic nematodes is limited [81,82]; because metam sodium undergoes rapid decomposition in moist soils to the active compound methyl isothiocyanate [83], soil fumigation of vegetable crops with metam sodium or metam potassium results in inconsistent control, particularly against root-knot nematodes, while intensive experience indicates its efficiency toward migratory plant-parasitic nematodes but no effect on root-knot nematodes. (iii) Dimethyl disulfide (DMDS), which was registered in the last decade and is effective at controlling both sedentary and migratory nematodes, as well as weeds and soilborne fungal pathogens. Unfortunately, the performance of these three fumigants is inferior to that of methyl bromide. In Israel, the prevalent treatment for nematode management in vegetables is targeted to reducing nematode population density primarily through soil fumigation with 1,3-D or DMDS. However, these fumigants do not provide adequate protection of crop health throughout the entire growing season. Therefore, an integrated approach is needed to achieve successful management of lesion nematodes.

### 7.2. Common Control *Methods in Used to Manage Plant Parasitic Nematodes*

**Figure 5:**
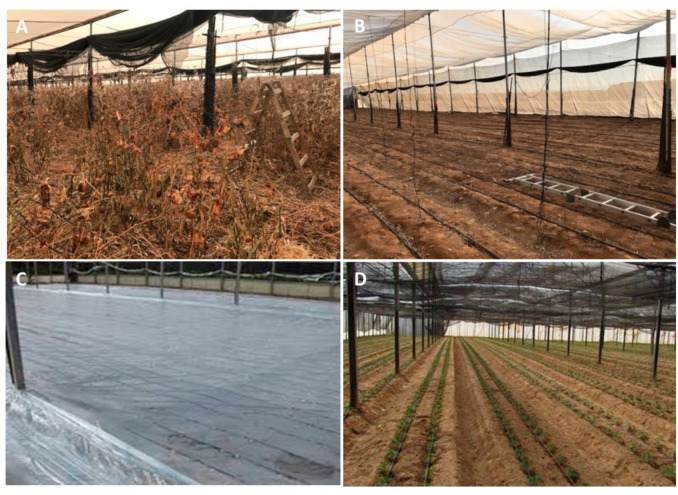
Integrated nematode management. Protocol used in practice to control migratory or sedentary plant-parasitic nematodes. (**A**) Destruction of previous crop’s roots before removal to reduce primary inoculum. (**B**) Root removal, tilling, and soil preparation for fumigation and solarization requirements. (**C**) Soil-disinfection approaches using different soil fumigants in combination with soil solarization for at least 4 weeks during the summer. (**D**) Planting of seedlings and reinstallation of shade net.

Currently recommended soil disinfestation approaches against soilborne plant-parasitic nematodes in conventional farming—mainly *Pratylenchus* species and *Meloidogyne* species root-knot nematodes—include the following steps [84]: (i) destruction of the plant roots at the end of the crop season before plant removal (Figure 5A); (ii) plant and root removal followed by tillage, although this latter recommendation is not always followed (Figure 5B); (iii) soil disinfection approaches using effective soil fumigants combined with soil solarization for a minimal period of 4 weeks during the summer (Figure 5C). At this time, nets above protected houses are removed to increase soil solarization efficiency, and shade nets are then reinstalled at seedling planting time (Figure 5D).

A combination of solarization with organic material (biosolarization) can reduce nematode densities but not achieve full eradication [85]. Similarly, Oka and Pivonia [86] explored the possibility of using ammonia for controlling soilborne diseases under variable environmental conditions in the Arava region of Israel. Given that soil pH may be the most important factor affecting the nematicidal activity of ammonia, where alkaline soils support better activity [87], as well as the fact that neutral to weakly alkaline sandy soils are common in Israel, the use of ammonia for nematode control is promising [86]. As expected, the use of NH_4_OH (at 500 and 1000 kg N/ha) increased tomato yield and reduced the galling index (at 1000 kg N/ha). However, despite its positive control effect, a high percentage of ammonia may be deleterious to the environment. This needs to be further evaluated under different soil conditions, nematicidal activities, and application methods. Another approach to exploiting ammonia for nematode control is the application of ammonia generators such as chicken manure, soy bean meal, and other organic materials [88]. Further studies by Oka et al. [89] demonstrated that application of ammonium sulfate, chicken litter and chitin, or neem (*Azadirachta indica*) extract alone failed to reduce the root galling index of tomato plants, but application of the amendments in combination with the neem extract reduced root galling significantly. Soil analysis indicated that the neem extract inhibits the nitrification of the ammonium released from the amendments and extends the persistence of the ammonium concentrations in the soil. In addition, biosolarization using chicken compost resulted in effective control of root-knot nematodes in a lettuce crop [88].

Field crops that are not under intensive production pose a challenge for nematode management. Orion et al. [10] found that leaving the soil fallow for 2 years reduced the *P. mediterraneus* population by 90% and increased wheat grain yields by 40–90%. By monitoring a 30-year rotation trial over several seasons of wheat-cropping systems, researchers found that the use of legumes (vetch, lentil) can increase *P. thornei* populations, whereas sunflower or safflower followed by a fallow period provided the best reduction of *P. thornei* [90]. Alternatively, soil treatment with metam sodium controlled *P. mediterraneus* by 90% and increased yield by 50–70% [91]. The biannual fallowing system was the most desirable environmentally, but it occupied 50% of the land, which in practice is problematic because cultivated land is quite limited in Israel. Since metam sodium treatment is less feasible in dryland agriculture, several alternative control methods were evaluated. Those studies suggested that nitrogen fertilizer does not affect *P. mediterraneus* populations in either dry farming or as a supplement in irrigation treatments [10]. Use of the nematicide formulation of furathiocarb, a systemic soil insecticide, as a seed dressing could reduce *P. mediterraneus* population level and increase yield, while the best nematode killing was achieved by soil application [11,14].

### 7.3. Resistance to Root-Lesion Nematodes

The wide host range of *Pratylenchus* species, and the restrictions, cost, and inefficiency of chemical nematicides have raised the importance of developing resistant cultivars as a control measure [92,93]. Unfortunately, only a few studies have considered the effects of resistance on *Pratylenchus* biology. Talavera and Van Stone [94] demonstrated that *P. thornei* is able to penetrate resistant cultivars. Farsi [95] observed equal root penetration by *P. neglectus* in both resistant and susceptible wheat lines. Other studies in various plant hosts have shown that, in other *Pratylenchus* species, resistance is associated with reduced motility and reproduction [96]. While the major studies of resistance have focused on wheat varieties [5], vegetable crops have been less investigated. The use of resistant cultivars is advantageous in integrated control programs because an accurate assessment of nematode infestations and infections is critical for the evaluation of plant resistance and tolerance to *Pratylenchus* species.

## 8. Challenges and Perspectives for *Pratylenchus* Research in Israel

In the last decade, several studies have been implemented toward the development of an integrated nematode management system that includes available and efficient means. Like elsewhere, most soil fumigants and nematicides belonging to containing organophosphates and carbamates have been withdrawn from the market or have strict use restrictions, mainly for environmental and safety reasons [97]. In general, there appears to be little prospect for the management of nematodes in many susceptible crops without repeated application of nematicides, which is economically justified in only a few cases. Alternatively, a number of products and formulations of fumigant–nematicides are available for use [98]. However, the effectiveness of traditional fumigants and nematicides with broad biocidal activity is declining, and the development of new classes of nematicides with novel activity and specific pest targets is perhaps an idealistic pipe dream. Recent research carried out in Israel has shown that the incorporation of nematicidal fluensulfone into the soil can reduce the populations of several migratory nematodes under laboratory conditions [44]. An additional new nematicidal compound, fluopyram, has been evaluated in vitro against root-knot nematodes [99], but its effect on migratory nematodes has not yet been confirmed in the field.

### 8.1. Taxonomy and Diagnosis of Pratylenchus Species

Given the wide distribution and severe damage caused by *Pratylenchus*, its taxonomy and diagnosis are crucial for *Pratylenchus* research and agricultural production in Israel. Despite its importance, the morphological diagnosis is greatly hampered by phenotypic plasticity, interspecific similarities, and a lack of molecular taxonomy specialists. Today, routine plant-parasitic nematode identification is conducted by the PPIS of the Israeli Ministry of Agriculture and Rural Development using only diagnostic morphological characteristics. The information provided to farmers, agronomists, nurseries, and inspectors consists mostly of identification at the genus level and the density of the nematode population found in the soil or root samples. Similarly, identification of *Pratylenchus* species is limited in most instances to the genus level, while species identification relies on the host from which they were recovered. Thus, molecular barcoding is a powerful, efficient, and reliable tool to simplify and standardize nematode identification, but such a method is not yet fully established for routine identification of *Pratylenchus* species, especially for basic research stations and production departments. Further effort is needed to expand *Pratylenchus* diagnostic techniques and improve farms’ awareness of them.

### 8.2. Control/Management of Pratylenchus Species

Extensive research is being performed on alternative chemical and nonchemical methods for controlling nematode diseases. However, these methods are generally less effective than soil fumigation in reducing soil nematode densities, and many have not proven consistent enough when used in intensive crop farming. Long-term field trials comparing the nematicide efficacies of several soil disinfestation methods would provide valuable information for nematode management. New nematicides are continually being introduced to the market although their efficiency against *Pratylenchus* species is not always known, and if it is, their label should refer to specific hosts, soils, and environmental conditions. Thus, the participation of professional nematologists is crucial in laboratory and field experiments evaluating nematicides. Symptoms caused by *Pratylenchus* species are frequently overlooked and a lack of nematological knowledge might lead to erroneous interpretations. Moreover, the migratory endoparasitic lifestyle, which might support the association of additional plant pathogens, should be studied for each plant–*Pratylenchus* interaction. In such cases, control strategies need to target both the nematode and the associated pathogen. A study of the etiology underlying nematode survival between seasons under extreme conditions is required to address important questions regarding the occurrence of anhydrobiosis, migration ability to lower soil levels, and factors required for these nematodes’ recovery. Exploration of these aspects is expected to contribute to the development of efficient integrated control management of *Pratylenchus*.

## 9. Conclusions

Delimitation of the various *Pratylenchus* species is considered to be very complicated, especially because of the small number of diagnostic features available at the species level and the intraspecific variability of some of these characteristics [23]. Nevertheless, due to the difficulty in separating species, the number of new proposed species of *Pratylenchus* has increased almost linearly, with a slope of 1.1 species per year between 1940 and 2006 [23]. Although morphology continues to be the basis for identification of *Pratylenchus* species, new technologies based on biochemical and molecular analyses are becoming increasingly important for nematode systematics and practical diagnoses [27,100,101,102]. New species are continuously being described through extensive morphological and molecular studies of the 28S D2-D3 expansion domains and ITS. The highest biodiversity of the genus is found in Asia, where 40 species have been reported, followed by Europe with 32, North America with 27, Central and South America with 22, Africa with 16, Oceania with 12, and Antarctica with a single species. The most widely distributed and common species are *P. neglectus*, *P. penetrans*, *P. thornei*, and *P. vulnus*, which have been reported on every continent with the exception of Antarctica. Thirty-seven species (54% of the 68 nominal species) in the genus have only been reported from a single continent, while the remaining 31 species (46%) have been reported from two or more continents. Nevertheless, despite the global distribution of the genus, some 32 of the described species have thus far only been recorded from their type locality. Along these lines, it will be interesting to determine whether, similar to *P. mediterraneus*, which was first found in Israel and later in other Middle Eastern countries, the occurrence of *P. capsici* will be identified in neighboring countries as well.

## Figures and Tables

**Table 1:** Percentage occurrence of plant-parasitic nematode genera in the rhizosphere of plant groups (genera occurring in 20% or more of samples) covering natural vegetation and cultivated crops in Israel [7].

Natural Vegetation					
Fruit Trees (*n* = 34)		Forest Trees (*n* = 20)		Herbaceous Plants (*n* = 12)	
*Tylenchorhynchus*	65	*Tylenchus*	70	*Helicotylenchus*	58
*Xiphinema*	62	*Xiphinema*	65	*Tylenchus*	58
*Helicotylenchus*	53	*Helicotylenchus*	60	*Meloidogyne*	50
*Tylenchus*	50	*Tylenchorhynchus*	40	*Tylenchorhynchus*	42
*Pratylenchus*	35	*Pratylenchus*	30	*Xiphinema*	33
*Meloidogyne*	29	*Rotylenchulus*	20		
*Rotylenchulus*	21				
**Cultivated crops**					
**Deciduous Fruit Trees (*n* = 38)**		**Subtropical and Tropical Fruit Trees (*n* = 20)**		**Grapevines (*n* = 17)**	
*Xiphinema*	76	*Xiphinema*	60	*Helicotylenchus*	65
*Tylenchorhynchus*	58	*Tylenchorhynchus*	55	*Xiphinema*	59
*Pratylenchus*	47	*Helicotylenchus*	45	*Tylenchorhynchus*	53
*Helicotylenchus*	38	*Tylenchus*	25	*Longidorus*	47
******		*Pratylenchus*	20	*Meloidogyne*	47
		*Criconemoides*	20	*Pratylenchus*	29
**Vegetable crops (*n* = 41)**		**Cereal crops and pasture grasses (*n* = 50)**		**Pasture Legumes (*n* = 27)**	
*Tylenchorhynchus*	59	*Tylenchorhynchus*	84	*Tylenchorhynchus*	85
*Pratylenchus*	49	*Pratylenchus*	68	*Tylenchus*	52
*Tylenchus*	39	*Tylenchus*	58	*Pratylenchus*	48
*Helicotylenchus*	27	*Helicotylenchus*	26	*Helicotylenchus*	30
*Longidorus*	22	*Xiphinema*	24	*Ditylenchus*	20
**Lawns (*n* = 11)**					
*Helicotylenchus*	82	*Criconemoides*	36	*Pratylenchus*	27
*Tylenchorhynchus*	64	*Xiphinema*	36		
*Tylenchus*	55	*Trichodorus*	27		

**Table 2:** Recent identification of plant-parasitic nematodes in several cultivated crops in Israel, recorded during 2018–2019, according to internal transcribed spacer (ITS).

Cultivated Crops					
**Grapevines**					
*Helicotylenchus pseudorobustus*	Tomer				
*Xiphinema index*	Tomer				
*Aphelenchoides* sp.	Tomer				
**Vegetable Crops**		**Cereal Crops and Pasture Grasses**		**Pasture Legumes**	
*Pratylenchus mediterraneus*	Kfar Silver	*Pratylenchus thornei*	Gevim	*Pratylenchus thornei*	Shif’a Gevim, NirOz
*Pratylenchus nanus*	Shoval	*Merlinius nanus*	Sde Eliyahu, Tirat Zvi	*Merlinius nanus*	Shif’a
*Pratylenchus thornei*	Shoval, Alumim	*Heterodera avenae*	Nirim	*Tylenchorhynchus clarus*	Nir David
*Neodolichorhynchus sulcatus*	Arava	*Heterodera sp.*	Nirim	*Rotylenchus macrosoma*	Shif’a
*Pratylenchus capsici*	Arava	*Geocenamus brevidens*	Nirim	*Tylenchorhynchus zeae*	Shif’a
*Meloidogyne javanica*	Mivtachim				

## Supplementary Materials

The following are available online at https://www.mdpi.com/2223-7747/9/11/1475/s1, Table S1: Morphometrics of *Pratylenchus* species reported from Israel. Reference [103] is cited in Table S1; Material File S2: Matrix Key Codes for the identification of *Pratylenchus* spp. according to Castillo and Vovlas.
