# Supplementary material for: The Genus Pratylenchus (Nematoda: Pratylenchidae) in Israel: From Taxonomy to Control Practices
Source: Plants (Basel). 2020 Nov 2;9(11):1475. doi: 10.3390/plants9111475 (PMC7716202; doi:10.3390/plants9111475)
Supplement: Supplementary file 1 [file plants-09-01475-s001.zip › Supplementary Table 1.docx]

**Supplementary Table 1.** Morphometrics of *Pratylenchus* species reported from Israel. Measurements and ratio ranges following Loof ^102^ and Qing et al.^18^ are indicated as female/male. Abbreviation of species names: **PM** = *P. mediterraneus*, **PT** = *P. thornei*, **PN** = *P. neglectus*, **PPR** =*P. pratensis*, **PV** = *P. vulnus*, **PC** = *P. capsici*, **PPE** = *P.* *penetrans*, **PS** = *P. scribneri*, **PB** = *P. brachyurus*.

| Species | a | b | c | V | Stylet (μm) | L (μm) |
| --- | --- | --- | --- | --- | --- | --- |
| **PM** | 24-31/27-35 | 5.2-7.6/5.6-6.3 | 17-25/18-22 | 77-80 | 14-16/13-15 | 428-577/431-542 |
| **PT** | 25.3-36.4/29.0 | 5.4-8.3/6.2 | 18.6-25.1/20.3 | 74.4-79.0 | 15-19/16 | 408-708/492 |
| **PN** | 16.5-32.2/25.5-28.9 | 4.9-7.8/6.2-6.3 | 13.8-26.8/17.3-21.7 | 75.5-86.6 | 15-19/15-17 | 312-588/420-524 |
| **PV** | 27.8-37.6/32.1-38.5 | 5.7-7.7/6.3-7.2 | 18.4-24.7/20.5-23.6 | 77-82 | 13-16/13-15 | 470-718/489-606 |
| **PPR** | 22.3-33.0/24.3-32.8 | 5.0-6.4/4.7-6.5 | 12.8-22.0/15.6-20.1 | 75-80 | 12-16/12-15 | 386-614/379-535 |
| **PC** | 22-30/25-35 | 5.3-8.1/6.1-7.5 | 17-24/19-26 | 70-81 | 14-15/13-15 | 559-642/530-598 |
| **PPE** | 19-32/23-34 | 5.3-7.9/5.4-7.3 | 15-24/16-22 | 75-84 | 15-17/13-16 | 350-810/310-570 |
| **PS** | 20-29/27.6 | 5.7-7.7/6.6 | 13.3-18.4/17.6 | 73-79 | 14-17/12 | 409-616/469 |
| **PB** | 15-29/27-29 | 5-10/6 | 13-28/21 | 82-89 | 17-22/19 | 393-750/460-560 |

^a^ Measurements and ratio ranges following Loof ^102^/Qing et al. ^18^
