# Supplementary material for: The Genus Pratylenchus (Nematoda: Pratylenchidae) in Israel: From Taxonomy to Control Practices
Source: Plants (Basel). 2020 Nov 2;9(11):1475. doi: 10.3390/plants9111475 (PMC7716202; doi:10.3390/plants9111475)
Supplement: Supplementary file 1 [file plants-09-01475-s001.zip › Supplementary material 2 PB.docx]

**Supplementary Material 2.** Matrix Key Codes for the identification of *Pratylenchus* spp according to Castillo and Vovlas [23].

**A) Lip annuli.**

**Group 1:** two

**Group 2:** three

**Group 3:** four

**B) Male.**

**Group 1:** absent

**Group 2:** present

**C) Stylet length**:

**Group 1:** stylet < 13 µm

**Group 2:** stylet 13-15.9 µm

**Group 3:** stylet 16-17.9 µm

**Group 4:** stylet 18-20 µm

**Group 5:** stylet > 20 µm

**D) Shape of spermatheca:**

**Group 1:** absent or reduced

**Group 2:** rounded to spherical

**Group 3:** oval

**Group 4:** rectangular

**E) Vulva position, ratio V:**

**Group 1:** V < 75%

**Group 2:** V = 75-79.9%

**Group 3:** V = 80-85%

**Group 4:** V > 85%

**F) Post-vulval uterine sac (PUS):**

**Group 1:** < 16 µm

**Group 2:** 16-19.9 µm

**Group 3:** 20-24.9 µm

**Group 4:** 25-29.9 µm

**Group 5:** 30-35 µm

**Group 6:** > 35 µm

**G) Female tail shape**:

**Group 1:** cylindrical

**Group 2:** subcylindrical

**Group 3:** conoid

**H) Female tail tip**:

**Group 1:** smooth

**Group 2:** striated

**Group 3:** pointed

**Group 4:** with ventral projection

**I) Pharyngeal overlapping length:**

**Group 1:** < 30 µm

**Group 2:** 30-39.9 µm

**Group 3:** 40-50 µm

**Group 4:** > 50 µm

**J) Lateral field lines at vulval region**:

**Group 1:** four

**Group 2:** five

**Group 3:** six to eight

**K) Lateral field structure at vulval region**

**Group 1:** smooth bands

**Group 2:** partially or completely areolated bands
